# Computational models as predictors of HIV treatment outcomes for the Phidisa cohort in South Africa

**DOI:** 10.4102/sajhivmed.v17i1.450

**Published:** 2016-06-30

**Authors:** Andrew Revell, Paul Khabo, Lotty Ledwaba, Sean Emery, Dechao Wang, Robin Wood, Carl Morrow, Hugo Tempelman, Raph L. Hamers, Peter Reiss, Ard van Sighem, Anton Pozniak, Julio Montaner, H. Clifford Lane, Brendan Larder

**Affiliations:** 1 The HIV Resistance Response Database Initiative (RDI), London, United Kingdom; 2 Project PHIDISA, South African Military Health Service (SAMHS), Lyttelton, South Africa; 3 Project PHIDISA, South African National Defence Force (SANDF), Lyttelton, South Africa; 4 Kirby Institute, University of New South Wales, Sydney, Australia; 5 The Desmond Tutu HIV Centre, University of Cape Town, South Africa; 6 Ndlovu Care Group, Elandsdoorn, South Africa; 7 Academic Medical Center of the University of Amsterdam, Amsterdam, the Netherlands; 8 Stichting HIV Monitoring, Amsterdam, the Netherlands; 9 Chelsea and Westminster Hospital, London, United Kingdom; 10 BC Centre for Excellence in HIV/AIDS, Vancouver, Canada; 11 National Institute of Allergy and Infectious Diseases, Bethesda, United States

## Abstract

**Background:**

Selecting the optimal combination of HIV drugs for an individual in resource-limited settings is challenging because of the limited availability of drugs and genotyping.

**Objective:**

The evaluation as a potential treatment support tool of computational models that predict response to therapy without a genotype, using cases from the Phidisa cohort in South Africa.

**Methods:**

Cases from Phidisa of treatment change following failure were identified that had the following data available: baseline CD4 count and viral load, details of failing and previous antiretroviral drugs, drugs in new regimen and time to follow-up. The HIV Resistance Response Database Initiative’s (RDI’s) models used these data to predict the probability of a viral load < 50 copies/mL at follow-up. The models were also used to identify effective alternative combinations of three locally available drugs.

**Results:**

The models achieved accuracy (area under the receiver–operator characteristic curve) of 0.72 when predicting response to therapy, which is less accurate than for an independent global test set (0.80) but at least comparable to that of genotyping with rules-based interpretation. The models were able to identify alternative locally available three-drug regimens that were predicted to be effective in 69% of all cases and 62% of those whose new treatment failed in the clinic.

**Conclusion:**

The predictive accuracy of the models for these South African patients together with the results of previous studies suggest that the RDI’s models have the potential to optimise treatment selection and reduce virological failure in different patient populations, without the use of a genotype.

## Introduction

The selection of a new combination of antiretroviral drugs when therapy fails in well-resourced countries is made on an individual basis using an extensive range of information that is at the physician’s disposal, usually including viral load values, CD4 counts, treatment history and, of particular relevance in the salvage situation, a viral genotype.^1,2^ Indeed, genotyping with interpretation by one of the many rules-based interpretation systems that are in widespread use is regarded by many as a foundation stone of individualised antiretroviral therapy, and has been demonstrated to be moderately predictive of virological response.^3,4^ Selecting the best combination of antiretrovirals in resource-limited settings with a limited range of drugs available and where a lack of funds, infrastructure and technical expertise make genotyping impractical, can be much more challenging.

In response to this challenge, the HIV Resistance Response Database Initiative (RDI) has developed computational models to assist in the selection of the most effective combinations of drugs from those available.^5,6^ The models are able to predict accurately virological response to combination antiretroviral therapy, with or without genotypic information, the latter basing their predictions on viral loads, CD4 counts, treatment history and time to follow-up.^7,8^

The RDI models are trained using longitudinal data from clinical cases where the HIV treatment has been changed and followed up. A case with all the necessary data (e.g. viral load and CD4 count at the time of the change, details of treatment history, drugs in new regimen, time to follow-up and follow-up viral load value) is termed a treatment-change episode (TCE). The models are trained using TCEs from the RDI database, containing data from 160 000 patients from more than 40 clinics, cohorts and clinical trials in more than 20 countries around the world in order to make the models’ predictions as generalisable as possible to patients from different settings. The models consistently achieve accuracy (measured as the area under the receiver operating characteristic curve [AUROC]) in the region of 75%–80% in their predictions of virological response to therapy.

The application of the models as a treatment decision-making aid has been assessed in prospective clinical pilot studies involving highly experienced HIV physicians in well-resourced settings and found to be a useful clinical tool.^9^ The RDI models are made freely available as a treatment-support tool, the HIV Treatment Response Prediction System (HIV-TRePS), via http://www.hivrdi.org.

In the EuResist versus Expert (EVE) study, the EuResist group has also reported the successful development of predictive models that performed as well as the predictions made by HIV physicians and virologists, albeit without the benefit of full treatment history, in a retrospective study.^10^

Historically the RDI models have been trained with data almost exclusively collected from well-resourced settings as this is where antiretroviral therapy was first available. While these models were highly accurate for cases from similar settings they were less so for cases from low-middle income countries not represented in the training data set, typically achieving AUROC values of 60%–70%.^7^ Nevertheless, this is comparable to the accuracy of using genotyping with rules-based interpretation to predict response to therapy. With the accelerated roll-out of HIV therapy in low middle-income countries, the RDI has now been able to collect significant data from such settings, predominantly sub-Saharan Africa, and has developed models including these data that have proved comparably accurate for test cases from high and low middle–income countries.^11^

Here we report on the evaluation of the current RF models used to power the online HIV-TRePS, using data from the Phidisa cohort in South Africa. Project Phidisa is a clinical research project focused on the management and treatment of HIV infection in the uniformed members of the South African National Defence Force (SANDF) and their dependents with HIV infection treated between 2004 and 2012.^12^ ‘Phidisa’ means ‘to heal’ in Setswana.

## Research design

### The models

The RF models used to power the HIV-TRePS system (V5.3.2.0) for patients without a genotype were trained to predict the probability of virological response (defined as plasma viral load < 50 copies HIV RNA/mL) to a new therapy introduced following virological failure (≥ 50 copies HIV-RNA/mL), using methods described in detail elsewhere.^6^ In the development of the models, the term ‘baseline’ relates to data collected while on the failing regimen. In summary, the following data from 22 567 TCEs including 1090 from Southern Africa were used: on-treatment baseline plasma viral load (sample taken ≤ 8 weeks prior to treatment change); on treatment baseline CD4 cell count (≤ 12 weeks prior to treatment change); baseline regimen (the drugs the patient was taking prior to the change); antiretroviral treatment history; drugs in the new regimen; a follow-up plasma viral load determination taken between 4 and 52 weeks following introduction of the new regimen and the time to that follow-up viral load. These data were coded as 42 input variables for input into the models: baseline viral load (log_10_ copies HIV RNA/mL); baseline CD4 count (cells/mm^3^); treatment history comprising 20 binary variables coding for any experience of zidovudine, didanosine, stavudine, abacavir, lamivudine, emtricitabine, tenofovir DF, efavirenz, nevirapine, etravirine, indinavir, nelfinavir, saquinavir, amprenavir, fos-amprenavir, lopinavir, atazanavir, darunavir, enfuvirtide, raltegravir; antiretroviral drugs in the new regimen, comprising 19 variables covering zidovudine, didanosine, stavudine, abacavir, lamivudine, emtricitabine, tenofovir DF, efavirenz, nevirapine, etravirine, indinavir, nelfinavir, saquinavir, (fos)amprenavir, lopinavir, atazanavir, darunavir, enfuvirtide, raltegravir; and time from treatment change to the follow-up viral load (number of days).^11^

The models’ performance was assessed by 10× cross-validation during model development and then with an independent global test set of 1000 cases including 100 from southern Africa, which were partitioned from the overall pool of available, complete TCEs. To prevent overfitting, we stopped the training process when the validation errors had their global minima. The accuracy of the models as predictors of virological response was evaluated using the models’ estimates of the probability of response following initiation of the new drug regimen and the actual responses observed in the clinic (binary response variable: response = 1 vs failure = 0) to plot ROC curves and assessing the AUROC. The optimum operating point (OOP) for the models that was derived during cross-validation was used as the cut-off for classifying predictions as ‘response’ or ‘failure’ and used to obtain the overall accuracy, sensitivity and specificity of the system. The models’ performance was compared with genotypic sensitivity scores derived from genotyping with rules-based interpretation systems (Stanford, ANRS and REGA), for those cases with genotypes available. The models achieved AUCs of 0.79–0.84 (mean of 0.82) during cross-validation, 0.80 with the global test set and 0.78 with the southern African subset. The AUCs were significantly lower (0.56–0.57) for genotyping with rules-based interpretation.^11^

TCEs were extracted from the full Phidisa data set that had all the data required by the models, as described above. The performance of the models as predictors of virological response for these cases was evaluated by comparing the average of the 10 RF models’ estimates of the probability of response following initiation of the new drug regimen to the actual responses observed in the Phidisa patients using the method described above.

### *In silico* analysis to identify effective alternative regimens

In order to assess the potential of the models to help avoid treatment failure in a resource-limited setting, where models that do not require a genotype may be of most value, they were used to identify antiretroviral regimens that were predicted to be effective for the Phidisa cases. Of particular interest were those cases where the new regimen selected in the clinic failed to re-suppress the virus. Baseline data were used by the models to make predictions of response for alternative three-drug regimens in common use, comprising only those drugs that were in use in the Phidisa cohort at the time. Again, the OOP (the cut-off above which the models’ estimate of the probability of a response is classified as a prediction of response) that was derived during model development was used, as a test of how generalisable the system is.

## Results

### Characteristics of the datasets

The baseline, treatment and response characteristics of the data sets are summarised in Table 1. The 402 Phidisa patients had somewhat lower baseline viral loads (median of 3.65 log_10_ copies/mL) than the global data used to train and test the models. The original test data set of 1000 TCEs had a median baseline viral load of 3.97 and the 100 southern African TCEs amongst them had a median viral load of 4.32. The median baseline CD4 count of the Phidisa cases was also somewhat lower at 230 cells/mL than the global data at 260 cells/mL but was substantially higher than the original 100 southern African TCEs at 163 cells/mL.

**TABLE 1:** Characteristics of the TCEs in the Phidisa and original test data sets.

Characteristics	Phidisa data	Original global independent test set †	Original southern African cases‡
**TCEs/patients**	402	1000	100
Male	189	661	36
Female	86	218	56
Not known	127	121	8
Median age (IQR)	35 (32–39)	39 (35–48)	35 (30–40)
**Baseline data**			
Median (IQR) baseline VL (log_10_ copies/mL)	3.65 (2.66–4.49)	3.97 (2.98–4.97)	4.32 (3.62–5.01)
Median (IQR) baseline CD4 (cells/mm^3^)	230 (139–328)	260 (123–387)	163 (65–362)
**Treatment history**			
No. switching 1st to 2nd line (%)	316 (79%)	381 (38%)	62 (62%)
No. switching 2nd to 3rd line (%)	55 (14%)	179 (18%)	20 (20%)
No. switching 3rd to 4th line (%)	23 (6%)	115 (12%)	11 (11%)
No. switching 4th line or beyond (%)	8 (2%)	325 (33%)	7 (7%)
Median no.(IQR) previous drugs	3 (3–3)	4 (3–6)	3 (3–4)
N(t)RTI experience (%)	402 (100%)	998 (100%)	100 (100%)
NNRTI experience (%)	360 (90%)	634 (63%)	94 (94%)
PI experience (%)	65 (16%)	630 (63%)	11 (11%)
**New regimens**			
2 N(t)RTI + PI (%)	198 (49.3%)	350 (35%)	70 (70%)
2 N(t)RTI + NNRTI (%)	141 (35.1%)	228 (23%)	22 (22%)
3 N(t)RTIs + PI (%)	2 (0.5%)	74 (7%)	2 (2%)
N(t)RTI + PI (dual therapy)	53 (13.2%)	10 (1%)	0
N(t)RTI + NNRTI (dual therapy)	4 (1.0%)	7 (0.7%)	0
2 N(t)RTI (dual therapy)	1 (0.25%)	23(2%)	2 (2%)
3 N(t)RTI + NNRTI	1 (0.25%)	40 (4%)	0
3 N(t)RTI + NNRTI + PI	1 (0.25%)	13 (1%)	0
4 N(t)RTI + NNRTI + PI	1 (0.25%)	4 (0.4%)	0
Other (%)	0 (0%)	251 (25%)	4 (4%)
Virological response (follow-up viral load < 50 copies/mL)	121 (30%)	364 (36%)	52 (52%)

† *n* = 1000;

‡ *n* = 100.

TCEs, treatment change episodes; IQR, interquartile range; VL, viral load; N(t)RTI, nucleoside or nucleotide reverse transcriptase inhibitor; NNRTI, non-nucleoside reverse transcriptase inhibitor; PI, protease inhibitor.

In other respects, as might be expected, the 402 Phidisa cases resembled the southern African subset of the original test set somewhat more than the global test set as a whole (and the training data from which they were partitioned). For example, 79% of the Phidisa patients were moving from their first-line to second-line therapy, as were 62% of the original South African test set, versus 38% of the global test set. The Phidisa and original Southern African cases had less previous drug exposure overall (median of 3 vs 4 drugs) and greater previous exposure to NNRTIs (90% and 94% vs 63%) and less to PIs (16% and 11% vs 63%), reflecting the fact that the great majority of the African cases were moving from first-line therapy of 2 N(t)RTI+NNRTI to second-line therapy, comprising 2N(t)RTI+PI in half of the Phidisa cases and 70% of the original southern African cases.

There was a similar proportion of virological failures amongst Phidisa cases (70%) and original training and test sets (66% and 64%) but fewer in the original southern African set (48%).

### Results of testing the models with the Phidisa treatment-change episodes

**FIGURE 1:**
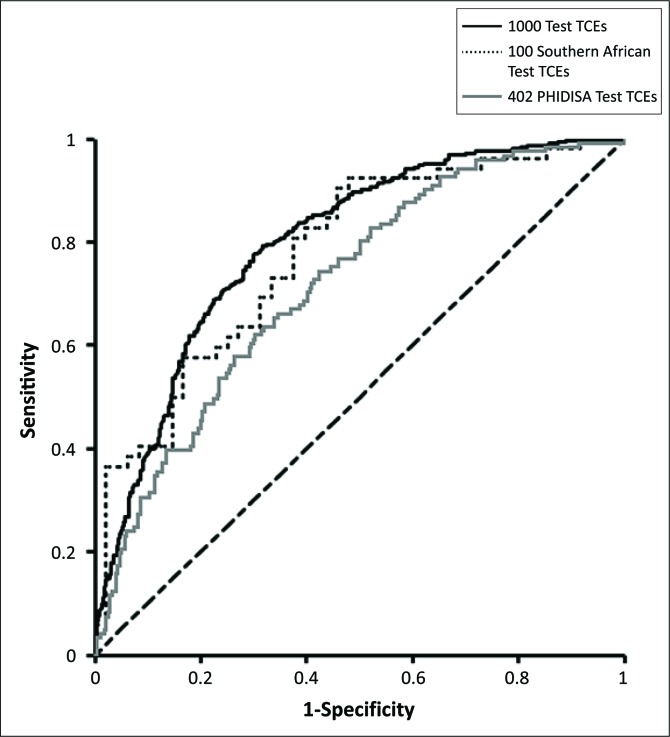
ROC curves for the committee of RF models tested with a global test set (*n* = 1000), the 100 southern African TCEs from that test set and the Phidisa cases (*N* = 402).

When tested using the Phidisa cases, the committee of 10 RF models achieved an AUC of 0.72, compared with 0.80 when tested with the original global test set and 0.78 with the 100 southern African TCEs within that test set (Table 2). The overall accuracy was 63% (vs 74% and 71% for the original test TCEs), the sensitivity 67% (vs 66% and 81%) and the specificity 62% (vs 79% and 60%). The ROC curve for the committee is presented in Figure 1. The difference between the performance of the models with the Phidisa cases and with the original global test set is statistically significant (*p* < 0.01), but the comparison with the 100 southern African TCEs within it was not (*p* = 0.26).

**TABLE 2:** Results of testing the models with the original independent test cases and the 402 Phidisa cases.

Variable	Phidisa cases†	Original test set‡	Original southern African TCEs§
Sensitivity	67%	66%	81%
Specificity	62%	79%	60%
Overall accuracy	63%	74%	71%
Statistical significance versus Phidisa	-	*p* < 0.01	*p* = 0.26 (ns)
Area under the ROC curve (AUC)	0.72	0.80	0.78

† *n* = 402;

‡ *n* = 1000;

§ *n* = 100.

When the models were tested separately with the cases of switching from first-line to second-line (*n* = 349) and later switches (*n* = 59), the results were not significantly different. The AUC values were 0.73 and 0.68 with overall accuracy of 64% and 68%, respectively.

### *In silico* analysis

The models were able to identify one or more three-drug regimens, comprising only those drugs present in the Phidisa database, that were predicted to be effective (the estimated probability of the follow-up viral load was above the OOP derived during cross validation), for 69% of all the Phidisa cases (Table 3). In these cases, the median number of alternative ‘effective’ regimens identified was 12. The models identified alternatives with a higher estimated likelihood of response than the regimen actually used in the clinic, but not necessarily above the OOP, in all 402 cases, with a median of 7 alternative regimens.

**TABLE 3:** *In silico* modelling to identify potentially effective alternative regimens for the Phidisa cases.

Variable	All cases†	Failures ‡ (70%)
Percentage of cases for which alternative three-drug regimens were predicted to be effective	69	62
Median number of alternatives	12	10
Percentage of cases for which alternative three-drug regimens were predicted to be more effective than the regimen selected	100	100
Median number of alternatives	7	8

† *n* = 402;

‡ *n* = 281.

There were 281 Phidisa patients (70%) that went on to fail the new regimen introduced in the clinic. For these, the models were able to identify one or more locally available three-drug regimens that were predicted to be effective in 62% of cases. The median number of these alternative regimens identified was 10. The models identified alternatives with a higher estimated likelihood of response than the regimen actually used in the clinic in all of the failures with a median of eight alternatives.

## Discussion

The RDI’s computational models that do not require a genotype predicted virological response to a change in antiretroviral therapy following virological failure for patients from the Phidisa cohort with a level of accuracy that was at least comparable to that of genotyping with rules-based interpretation as a predictor of virological response, as observed in several previous studies.^3,4,11^ The AUC value of 0.72 for the models compares favourably with AUC values typically in the range of 0.55–0.65 for genotyping. Genotyping is not used to select the next regimen in South Africa but mainly to differentiate patients with drug resistance from those with poor adherence and detect patients with second-line failure who require a third-line, selected on the basis of clinical information and according to strict protocols as part of a public health strategy. Nevertheless, genotyping with rules-based interpretation is widely considered as the gold standard for predicting drug sensitivity and response and is in widespread use for individualised treatment selection around the world. As such it provides a highly relevant benchmark against which to compare this and other approaches for predicting response to HIV therapy.

It is also encouraging that the models were able to identify several alternative, available three-drug regimens that were predicted to produce a virological response for two-thirds of the cases from the Phidisa cohort, including the virological failures. Furthermore, the models were able to identify regimens with a higher predicted probability of success than the regimen that failed in the clinic, for all cases.

The online treatment support tool, HIV-TRePS, through which the models are made available has the facility for users to include the annual cost of drugs in their setting and instruct the system to include the annual costs of different regimens in the report, alongside the predictions of response produced by the models. This raises the possibility that physicians can use the system to identify alternative regimens that are not only predicted to be more likely to produce a response but may be less costly than the regimen they would otherwise use. Indeed, a recent study of cases treated in India revealed that substantial cost savings may be possible through use of the system.^13^

The models’ accuracy of prediction for the Phidisa cases was somewhat less than that observed during model development and previous independent testing with cases from a range of settings, including a subset from southern Africa, although the latter difference did not achieve statistical significance. South Africa has a uniform programme with strict and rational criteria for regimen switches. In this context, the moderate performance of the TRePS algorithm is not unexpected.

When tested with the original global test set, using the OOP derived during cross-validation, the models showed higher specificity at 79% than sensitivity (66%). This is the pattern found in previous modelling studies and means the models are generally ‘conservative’, making relatively fewer incorrect predictions of response than incorrect predictions of failure. It is interesting to note that the reverse was true when the models were tested with the subset of the original test set that came from southern Africa (specificity of 60% and sensitivity of 81%). This suggests that an upward adjustment of the OOP above which a prediction would be classified as a response might be desirable to rebalance the classification and optimise performance for patients from this region, possibly related to treatment starting later in the course of the disease than for the global data. For the Phidisa cases, the specificity was again reduced at 62%, meaning that the models incorrectly predicted response for 38% of the observed failures. However, unlike the original southern African test cases, there was no apparent compensatory increase in sensitivity, which was 67%.

The fact that the Phidisa patients had somewhat lower baseline viral loads than the original global data used to train and test the models, as well as the southern African cases from the original test set, and substantially higher CD4 counts than the latter is consistent with Phidisa being a closely monitored cohort and much of the training data being collected from open clinical practice. Nevertheless, the Phidisa and the original southern African patients were mainly moving from first- to second-line therapy, so the lower sensitivity of the models (60%) and lower observed response rate (30%) for the Phidisa patients compared with the original southern African patients (81% and 52%, respectively) remains unexplained.

It is important to note that one of the input variables for these models was the plasma viral load, which previous studies have shown to be very important to the predictive accuracy of the models.^14^ Although viral load monitoring is not yet routine in most resource-limited settings, it is now recommended as the preferred approach to monitoring antiretroviral therapy success and diagnosing treatment failure in the latest WHO guidelines.^15^ As technological advances enable lower test costs and simpler equipment requiring less infrastructure, maintenance and technical expertise, so the use of viral load in clinical practice is likely to increase.^16^ It should also be noted that another input variable is the CD4 count. This is more affordable and in more widespread use in resource-limited settings but not universal, and its use may diminish as viral load testing expands.

The definition of virological failure used in the study was a single viral load value of > 50 copies HIV RNA/mL, compared with repeated measure of 400 copies/mL or 1000 copies/mL in clinical practice in South Africa. This threshold was used because the majority of the data used to train the models and the majority of the settings in which the models are used use a definition of 50 copies/mL. A single measure was used in order to maximise the number of TCEs available for training the models. As the size of the RDI data set increases, we could consider the use of multiple viral load measurements and the exclusion of those cases with only one.

The study has some limitations. Firstly, it was retrospective and, as such, no firm claims can be made for the clinical benefit that the use of the system as a treatment support tool could provide. Another limitation is that the Phidisa cases came from a carefully monitored, military cohort and the cases used in the analysis are, by definition, those with complete data around a change of therapy. Such data may not be truly representative of the general patient population. Nevertheless, the performance of the models in predicting outcomes for this independent South African cohort is encouraging in terms of the applicability of the approach.

The RDI models and more accurately the HIV-TRePS system that they power have wide-ranging potential utility in South Africa and other resource-limited settings, for example:

- In switching from first- to second-line, following treatment protocols, the system can provide predictions of which NRTI backbone and choice of third agent, comprising locally available drugs, offer the highest probability of response.
- In switching from second to third-line or beyond, the system can help the healthcare professional assemble an individualised regimen with the highest probability of response.
- In doing so, the system can utilise genotypic information where available, or produce predictions of response that are comparable, or are most likely superior to those of genotyping with rules-based interpretation for cases where genotyping is not available or affordable.
- The system can help to reduce treatment costs. By entering their local drug costs into the system, healthcare professionals can identify the most effective regimens within a budget limit, or select the least costly of a number of regimens with similar estimates of effectiveness.
- By putting the distilled treatment experience of hundreds of physicians treating tens of thousands of patients around the world at their fingertips, the system can give relatively inexperienced healthcare professionals the confidence to make treatment decisions in settings or cases not covered by current treatment guidelines, for example, in salvage therapy with limited drug options available.

## Conclusion

In this study, we challenged the RDI models that do not require a genotype to predict virological response for patients in the Phidisa military cohort in South Africa, most of whom were moving from first- to second-line therapy. The models performed less well than with a more diverse global test set but still achieved a level of accuracy that was at least comparable to that observed in previous studies using genotyping with rules-based interpretation as a predictor of response.

It is encouraging that the models were able to identify alternative, available regimens that were predicted to be effective for the majority of the Phidisa cases, including those that failed the new regimen prescribed in the clinic.

These results and those of previous published studies suggest this approach has the potential to optimise treatment selection, reduce virological failure, improve patient outcomes and potentially reduce drug costs in South Africa and other resource-limited settings where resistance testing is unavailable or unaffordable.

Note: The methodology, development and cross-validation of the random forest models studied in this paper were described in previous publications by the RDI and its collaborators (6, 7). Consequently, there are some overlaps between parts of the Methods section of the papers. The novel aspect of the current paper is the evaluation of the models as a potential clinical tool with a substantial cohort of patients from South Africa.
